# Predictors of Post-Hepatectomy Liver Failure in Klatskin Tumors: The Role of Preoperative Glucose, Future Liver Remnant to Spleen Ratio, and Early Bilirubin Monitoring

**DOI:** 10.3390/diagnostics14232716

**Published:** 2024-12-02

**Authors:** Suyeon Kim, Hyung June Ku, Hyung Hwan Moon, Sang Hwa Song, Young Il Choi, Dong Hoon Shin, Yang Seok Koh, Namkee Oh, Jinsoo Rhu, Garam Lee, Won Jong Yang, Junho Song, Chol Min Kang, Seoyeong Ku, Amy Choi

**Affiliations:** 1 Department of Information Medicine, Asan Medical Center, University of Ulsan College of Medicine, Seoul 05505, Republic of Korea; ra02680@amc.seoul.kr; 2 Chang Kee-Ryo Memorial Liver Institute, Kosin University College of Medicine, Busan 49267, Republic of Korea; hjkoo1995@gmail.com (H.J.K.); tsojc@naver.com (Y.I.C.); surgeonshin@naver.com (D.H.S.); tpy3728@jnu.ac.kr (G.L.); ksy3124@swu.ac.kr (S.K.); 3 Division of Hepatobiliary-Pancreas and Transplantation, Department of Surgery, Kosin University Gospel Hospital, Busan 49267, Republic of Korea; 4 Division of Hepatobiliary-Pancreas Surgery, Department of Surgery, Chonnam National University Hwasun Hospital, Hwasun 58128, Republic of Korea; yskoh@chonnam.ac.kr; 5 Department of Surgery, Samsung Medical Center, Sungkyunkwan University School of Medicine, Seoul 06351, Republic of Korea; ngnyou@gmail.com (N.O.); jsrrules@gmail.com (J.R.); 6 College of Veterinary Medicine, Chonnam National University, Gwangju 61186, Republic of Korea; 7 Computer Science Department, Silver Center for Arts and Science, New York University, New York, NY 10010, USA; wjy220@nyu.edu; 8 College of Medicine, Pennsylvania State University, Hershey, PA 17033, USA; jsong4@pennstatehealth.psu.edu; 9 Department of Applied Biomedical Engineering, The Johns Hopkins University, Baltimore, MD 21287, USA; kcholmin@gmail.com; 10 Department of Horticulture, Life and Landscape Architecture, Seoul Women’s University, Seoul 01797, Republic of Korea; 11 Department of Neurobiology, Columbia University, New York, NY 10027, USA; ac4505@columbia.edu

**Keywords:** klatskin tumor, perihilar cholangiocarcinoma, post-hepatectomy liver failure, volumetric assessment, predictors of post-hepatectomy liver failure

## Abstract

**Background**: Post-hepatectomy liver failure (PHLF) is a serious complication following hepatic resection for Klatskin tumors, significantly affecting patient prognosis. Identifying reliable preoperative and early postoperative predictors of PHLF can help optimize patient outcomes and guide surgical planning. **Method**: We conducted a retrospective review of 34 patients who underwent hemi-hepatectomy for extrahepatic cholangiocarcinoma at Kosin University Gospel Hospital between April 2019 and April 2024, and at Chonnam National University Hwasun Hospital between September 2017 and April 2024. Demographics, laboratory data, and volumetric measurements including spleen volume, were analyzed to assess their roles in predicting PHLF. Logistic regression and receiver operating characteristic (ROC) curve analyses were performed to evaluate the predictive value of these factors. **Results**: Elevated preoperative glucose levels and reduced future liver remnant to spleen ratio (FLR/SV) were significantly associated with an increased risk of PHLF. Additionally, elevated postoperative day 1 total bilirubin (POD 1 TB) was identified as a key postoperative predictor of PHLF. Multivariable analysis confirmed the significance of these factors, with FLR/SV, preoperative glucose, and POD 1 TB demonstrating good discriminative ability in ROC analysis, with AUC values of 0.779, 0.782, and 0.786 respectively. **Conclusions**: Preoperative glucose control, evaluation of FLR/SV, and early postoperative monitoring of TB are useful for improving outcomes in patients undergoing major hepatectomy for Klatskin tumors.

## 1. Introduction

Klatskin tumors, also known as perihilar cholangiocarcinoma, are a type of bile duct cancer located at the hepatic hilum, where the left and right hepatic ducts converge [1]. These tumors present diagnostic challenges due to their location and nonspecific symptoms, often resulting in late presentation. The primary treatment goal for Klatskin tumors is complete surgical resection, which remains the only potential cure for these patients. However, even with successful resection, the incidence of post-hepatectomy liver failure (PHLF) is significant, adversely affecting patient prognosis [2,3]. PHLF is a major complication following hepatic surgery and is associated with high morbidity and mortality rates, making it a critical factor in patient outcomes [4].

The high incidence of liver failure in patients undergoing surgery for Klatskin tumors underscores the need to better understand the associated risk factors. The causes of PHLF are multifactorial, often involving preoperative liver function, the extent of liver resection, and the regenerative capacity of the future liver remnant (FLR) [5,6]. Insufficient FLR volume, pre-existing liver disease, and impaired hepatic function have all been identified as significant predictors of PHLF, emphasizing the need for careful preoperative planning and risk stratification [7,8].

The causes of PHLF are multifactorial, often involving preoperative liver function, the extent of liver resection, and the regenerative capacity of the future liver remnant (FLR). Previous studies have consistently demonstrated that impaired preoperative liver function, as indicated by elevated bilirubin levels and reduced albumin levels, is a significant predictor of PHLF [9,10]. Insufficient FLR volume has also been widely acknowledged as a major risk factor for PHLF, largely due to the compromised regenerative ability of the liver when the residual volume is inadequate to maintain normal function [11,12]. Moreover, underlying liver diseases, such as fibrosis or cirrhosis, further exacerbate the risk of PHLF by reducing the regenerative capacity of hepatocytes [13]. Bucur et al. emphasized that portal hypertension, often linked with pre-existing liver conditions, significantly increases the risk of PHLF by altering hepatic hemodynamics and impairing regeneration [14]. Additionally, the extent of liver resection plays a critical role, as larger resections leave a smaller FLR, which is less capable of compensating for the lost hepatic mass [15]. Recent evidence suggests that preoperative spleen volume could serve as an important metric for assessing the risk of PHLF, as a disproportionate spleen volume may indicate underlying portal hypertension, which negatively affects liver regeneration [16]. These factors collectively highlight the importance of thorough preoperative evaluation, including liver function tests, volumetric analysis, and assessment of underlying liver disease, to mitigate the risk of PHLF and guide surgical decision-making.

Building on this understanding, this study aims to analyze the predictors of liver failure following surgery for Klatskin tumors and identify specific factors that can be used to improve patient outcomes. By investigating computed tomography (CT) volumetry and other preoperative factors, this study seeks to provide insights that may improve risk assessment and surgical planning. Unlike previous studies that primarily focused on liver function tests or volumetric analysis alone, this study introduces a combined approach, utilizing both liver and spleen dynamics to enhance predictive accuracy.

## 2. Methods

### 2.1. Patient Selection and Data Collection

**Figure 1:**
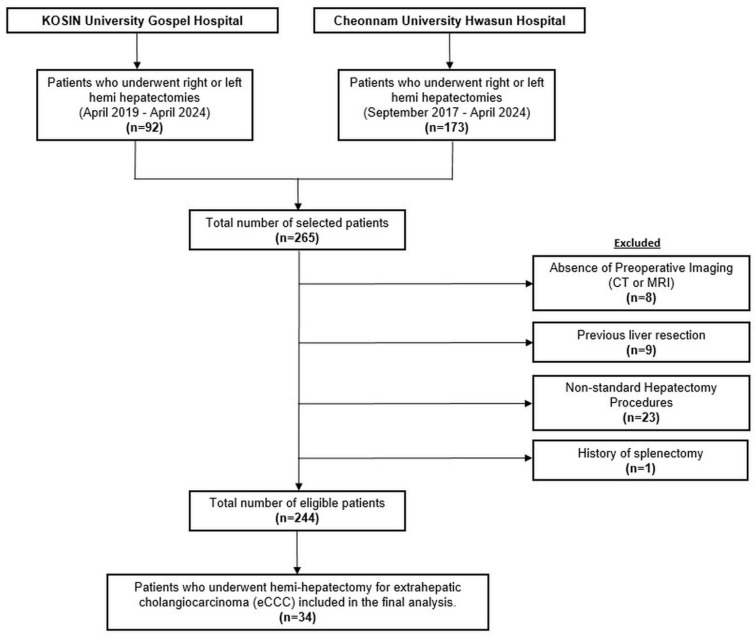
Flowchart of patient selection.

We conducted a retrospective review of consecutive patients who underwent major hepatectomy, including left or right hemi-hepatectomy, at Kosin University Gospel Hospital between April 2019 and April 2024, and Cheonnam National University Hwasun Hospital between September 2017 and April 2024. This study was approved by the Institutional Review Boards of Kosin University Gospel Hospital (KUGH 2024-08-029) and Chonnam National University Hwasun Hospital (CNUHH-2024-179). Patients with a history of prior hepatectomy, splenectomy, or lack of preoperative imaging (CT or MRI) were excluded. After applying these exclusion criteria, 34 patients who underwent hemi-hepatectomy for extrahepatic cholangiocarcinoma (eCCC) were included in the final analysis (Figure 1).

We collected preoperative demographic data, including age, sex, height, weight, body mass index (BMI), American Society of Anesthesiologists (ASA) classification, and underlying comorbidities such as hypertension, diabetes mellitus, viral hepatitis, and alcoholism (Table 1). Perioperative factors, such as the type of liver resection, operative time, and estimated blood loss, were also recorded. Laboratory characteristics, including platelet count, liver function tests, APRI, ALBI, and MELD scores, were collected and analyzed.

### 2.2. Computed Tomography Volumetry for Liver and Spleen

**Figure 2:**
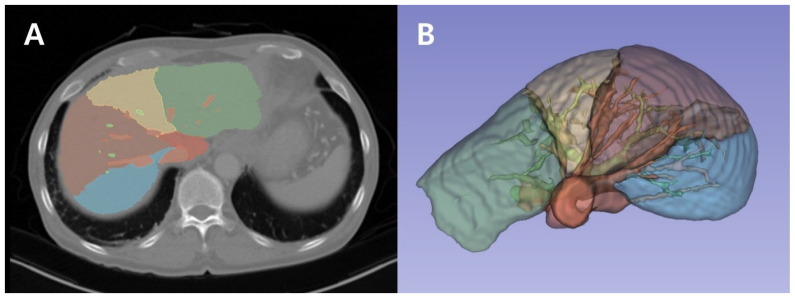
Liver segmentation using a 3D residual U-Net model. (**A**). Segmented liver from abdominal CT scan. Liver segments are color-coded to demonstrate regional anatomical structures, as viewed from the axial plane (horizontal cross-section). (**B**). 3D volumetric rendering of the liver. The liver lobes and associated vascular structures are segmented and color-coded to enhance clarity.

CT volumetry was performed using a 3D residual U-Net model, as described in the referenced literature. The model effectively captures context features through an encoding–decoding process [17,18,19]. During encoding, convolutional residual operations extract features, and during decoding, multi-target segmentation is achieved using deconvolutional feature maps, incorporating skip connections. The model was implemented in TensorFlow 1.14 and trained on a system equipped with four NVIDIA TITAN XP GPUs. Training was conducted using the Adam optimizer with a learning rate of 0.0001 over 1000 epochs. The Dice similarity coefficient was used as the loss function to optimize segmentation accuracy, and additional augmentation techniques, such as 3D rotation and scaling, were applied during training to improve model robustness (Figure 2).

**Figure 3:**
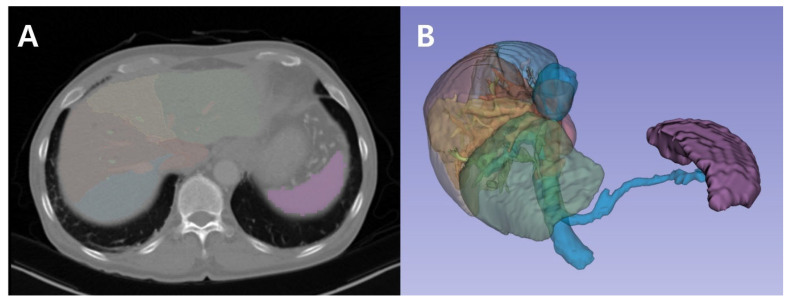
Spleen volumetry using 3D Slicer’s Total Segmentator. (**A**). Segmented spleen from abdominal CT scan. The spleen is highlighted in purple, with the liver faintly visible, to demonstrate their anatomical structures as viewed from the axial plane (horizontal cross-section). (**B**). 3D volumetric rendering of the spleen. The spleen is highlighted in purple, along with its associated vascular structures and connection to the liver.

Spleen volume was measured preoperatively using the 3D Slicer software (version 5.6.2; 2024-04-05; https://www.slicer.org/ (accessed on 30 November 2024), an open-source platform widely used for medical image analysis. Spleen segmentation was performed using the Total Segmentator extension, which employs deep learning algorithms for automated organ delineation. This tool leverages convolutional neural networks (CNNs) to accurately segment multiple organs, including the spleen, from CT images. Due to the built-in preprocessing capabilities of the Total Segmentator, no additional manual preprocessing steps were necessary. Segmentation accuracy was verified by overlaying the generated spleen mask onto the original CT images in axial, coronal, and sagittal views. Once the segmentation was confirmed to accurately capture the splenic contours, spleen volume was calculated using the Segment Statistics module in 3D Slicer. This module computes the total volume (in cubic centimeters, cm^3^) by aggregating the volumes of all segmented voxels within the mask. All measurements were reviewed by an experienced physician to ensure accuracy and consistency in the segmentation process (Figure 3).

### 2.3. Definition of Post-Hepatectomy Liver Failure

Post-hepatectomy liver failure (PHLF) was defined according to the criteria established by the International Study Group of Liver Surgery (ISGLS) [20]. The ISGLS criteria classify PHLF based on alterations in the international normalized ratio (INR) and bilirubin levels, which fail to return to normal within the first 5 postoperative days. The severity of PHLF is further categorized into grades A, B, and C, depending on the level of clinical management required: grade A requires no change in the patient’s management; grade B necessitates non-invasive management; and grade C requires invasive treatment or organ support. This standardized definition allows for consistent reporting and comparison of PHLF outcomes across different studies. We considered grades B and C as clinically significant PHLF.

### 2.4. Statistical Analysis

Continuous variables are presented as means to two decimal places (minimum–maximum values), while categorical variables are presented as frequencies with percentages (N, %). The Mann–Whitney U test was used for comparisons of continuous variables, and the chi-squared test was applied for categorical variables. To identify factors associated with liver failure, a logistic regression analysis was performed, incorporating clinically relevant variables and adjusting for potential confounders.

During the multivariate analysis, both preoperative and postoperative variables were initially included in the model. However, variables with a variance inflation factor (VIF) of 10 or higher were identified, indicating multicollinearity. To address this issue, preoperative and postoperative variables were analyzed separately in the multivariate analysis.

Statistical significance was defined as a *p*-value of less than 0.05. Receiver operating characteristic (ROC) curve analysis was performed to evaluate the diagnostic performance of identified factors, and the area under the curve (AUC) was calculated to assess the accuracy of the predictive models. All statistical analyses, including multivariate logistic regression, VIF calculations, and ROC curve analysis, were performed using a statistical modeling pipeline based on Python programming language (version 3.11). Specifically, the statsmodels library was utilized for logistic regression and VIF calculations, and the scikit-learn library was employed for ROC curve analysis and AUC calculations. All data processing and visualization were carried out using Python libraries, including pandas and matplotlib.

## 3. Results

Table 1 presents the preoperative demographics of 34 patients who underwent surgery for Klatskin tumors, comparing those without PHLF or with ISGLS grade A (*n* = 26) to those with ISGLS grade B or C (*n* = 8). The analysis revealed significant differences in preoperative glucose levels and FLR/SV ratios between the two groups. Specifically, preoperative glucose levels were higher in the ISGLS grade B/C group (*p* = 0.031), and FLR/SV ratios were lower (*p* = 0.012). In the multivariable logistic regression analysis, both preoperative glucose and FLR/SV were found to be significant predictors of PHLF, with *p*-values of 0.047 and 0.038, respectively (Table 2). These findings indicate that elevated preoperative glucose and reduced FLR/SV ratios are associated with an increased risk of developing PHLF, emphasizing the importance of metabolic control and careful preoperative volumetric assessment.

Table 3 provides an overview of the immediate postoperative demographics of the same patient cohort, comparing patients without PHLF or with ISGLS grade A to those with ISGLS grade B or C. Significant differences were observed in operative time, white blood cell counts on postoperative day 1 (POD 1 WBC), and POD 1 total bilirubin (TB). The ISGLS grade B/C group had a longer operative time (*p* = 0.043), lower POD 1 WBC (*p* = 0.011), and higher POD 1 TB (*p* = 0.006) compared to the ISGLS grade A/PHLF-negative group. In the multivariable analysis, only POD 1 TB remained a statistically significant predictor of PHLF, with an odds ratio of 5.821 (95% CI: 1.136–29.818, *p* = 0.035) (Table 4). This suggests that elevated POD 1 TB is an important early marker of liver dysfunction following major hepatectomy for Klatskin tumors.

**Figure 4:**
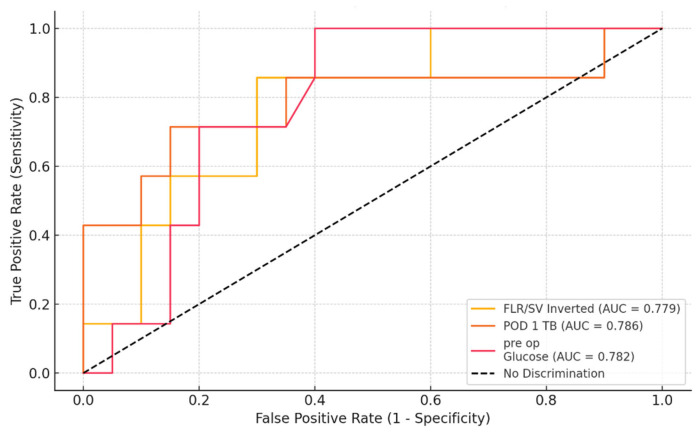
ROC curve analysis for predicting ISGLS B/C using future liver remnant to spleen ratio, postoperative day 1 total bilirubin, and preoperative glucose.

The ROC curve analysis further supports the predictive value of FLR/SV (inverted), POD 1 TB, and preoperative glucose for PHLF (Figure 4). All three variables demonstrated AUC values close to 0.8, indicating good discriminative ability. Specifically, FLR/SV (inverted) had an AUC of 0.779, POD 1 TB had an AUC of 0.786, and preoperative glucose had an AUC of 0.782. These findings suggest that each of these variables is a reliable predictor of PHLF, offering potential utility in preoperative and early postoperative risk stratification. The identification of these markers highlights the importance of both preoperative optimization and vigilant postoperative monitoring to improve patient outcomes following surgery for Klatskin tumors.

## 4. Discussion

In this study, we identified elevated preoperative glucose levels, reduced FLR/SV, and increased POD 1 TB as significant predictors of PHLF. Both preoperative glucose and FLR/SV were significant in multivariable analysis, suggesting their critical role in identifying patients at higher risk of PHLF. Our results emphasize the importance of metabolic control and volumetric assessment before surgery, as well as early monitoring of bilirubin postoperatively.

Elevated preoperative glucose levels were significantly associated with an increased risk of PHLF, which aligns with findings from previous research on hyperglycemia and liver regeneration. Specifically, Wang et al. demonstrated that hyperglycemia impairs the regenerative capacity of the liver by promoting a pro-inflammatory environment and increasing oxidative stress, which hinders hepatocyte proliferation and reduces liver regenerative potential [21]. Hyperglycemia not only adversely affects the hepatic microenvironment but also increases the risk of complications following major liver surgery [22]. Our findings are consistent with these previous reports, reinforcing the necessity of preoperative glucose control in patients undergoing liver resection for Klatskin tumors. By controlling glucose levels, the inflammatory response can be minimized, potentially improving liver regeneration and reducing the risk of PHLF.

It is well established that FLR volume is a key predictor of PHLF. Søreide et al. emphasized that insufficient FLR is associated with poor postoperative outcomes and an increased risk of liver failure due to the reduced capacity of the liver remnant to handle metabolic demands and regenerate effectively [23,24]. Our study corroborates these findings, with FLR being a significant predictor of PHLF. However, our study adds a new dimension by investigating the role of spleen volume in the risk stratification of PHLF. While the association between FLR and PHLF is well documented, there is limited literature on the impact of spleen volume as a risk factor for PHLF.

The spleen plays an important role in the body’s immune response and hematologic function, and its size can reflect underlying liver pathology. Yu al. discussed that splenomegaly is often observed in patients with liver fibrosis or cirrhosis, conditions that impair liver function and reduce the regenerative capacity of the liver [25]. Splenomegaly is typically caused by portal hypertension, a common consequence of advanced liver disease, where increased pressure in the portal vein leads to congestion and enlargement of the spleen. This enlargement of the spleen can serve as an indirect marker of the severity of liver disease. In addition, Romero et al. highlighted that increased spleen volume could indicate portal hypertension, correlating with increased surgical risk [26]. Similarly, Ito et al. identified the spleen volume/body surface area as a predictor of post-hepatectomy liver failure and short-term mortality in hepatocellular carcinoma [27].

Previous studies have demonstrated the association between spleen size or volume and liver condition, as well as postoperative liver failure. However, the extent of liver resection remains a critical determinant of postoperative liver failure in patients undergoing liver surgery. In this context, our finding that the FLR/SV ratio is a significant predictor of PHLF adds a meaningful dimension to risk assessment. Specifically, a lower FLR/SV ratio (median value: 2.87, range 1.12–6.01) was associated with a higher risk of liver failure. This finding suggests that spleen volume, in conjunction with FLR, can provide a more comprehensive assessment of liver function and the risk of PHLF, particularly in patients with underlying liver disease.

The finding that FLR/SV is a significant predictor of PHLF has important clinical implications. It suggests that not only should the volume of the future liver remnant be considered in preoperative planning, but spleen volume should also be taken into account. A larger spleen may indicate more advanced liver disease, reduced hepatic regenerative capacity, and increased portal pressure, all of which could increase the risk of liver failure after resection. Therefore, incorporating spleen volume measurement and calculating the FLR/SV ratio can enhance preoperative assessments, helping surgeons identify patients at higher risk of PHLF and adapt surgical strategies accordingly. This may include preoperative interventions such as portal vein embolization (PVE) to increase the FLR before surgery, improving the safety of the procedure [28]. Chan et al. discussed that portal pressure modulation through preoperative interventions like PVE or associating liver partition and portal vein ligation for staged hepatectomy (ALPPS) could also be a strategy to enhance FLR and minimize risks [29]. These interventions are especially relevant for patients with marginal FLR or those exhibiting splenomegaly and portal hypertension. Therefore, integrating spleen volume assessment into preoperative evaluation could facilitate more personalized and safer surgical approaches for high-risk patients. Moreover, preoperative imaging and volumetric analysis are suggested to be key in determining the optimal approach to mitigate risks related to insufficient FLR and spleen-related risk factors [30].

Another critical finding was the significant association between POD 1 TB levels and the risk of PHLF. Etra et al. identified POD 3 TB as an early marker of impaired liver function, reflecting the liver’s inability to adequately excrete bile following major hepatectomy [31]. Sawangkajohn et al. further showed that elevated postoperative bilirubin levels correlate with poor liver function and an increased risk of complications [32]. In our cohort, patients with higher POD 1 TB levels were more likely to develop PHLF, and this association remained significant in multivariable analysis. This underscores the importance of early postoperative monitoring of bilirubin levels to identify patients who may be at risk of liver failure, enabling timely intervention to prevent progression to more severe liver dysfunction.

These findings are consistent with previous literature, indicating that both metabolic factors and volumetric assessments are crucial for predicting liver failure following major hepatectomy. Unlike previous studies that primarily focused on either liver function tests or volumetric analysis alone, our study introduces a combined approach integrating both liver and spleen dynamics to enhance predictive accuracy [27,33]. In addition, with the emergence of machine learning in predicting PHLF, integrating the features identified in our study into prediction models could further enhance their accuracy [34].

This study has several limitations. First, its retrospective nature may have introduced bias in data collection and interpretation. Second, data were collected from two institutions, which may have introduced institutional biases. Third, the study’s relatively small sample size limits the generalizability of the findings. Additionally, several instances of missing preoperative glucose data were identified, which may have introduced bias in the results. The absence of postoperative glucose data, which were neither collected nor analyzed, further limits the findings of this study. Lastly, when applying the ISGLS criteria to PHLF, clinical judgment regarding symptoms may involve subjectivity, which could potentially affect the consistency of ISGLS A, B, and C classifications.

Despite these limitations, our findings suggest that managing hyperglycemia in the preoperative period could improve liver regenerative capacity and reduce the incidence of PHLF. Furthermore, FLR/SV assessment could be integrated into preoperative planning, providing a nuanced risk stratification method compared to FLR assessment alone. Monitoring immediate postoperative TB could also be crucial for early detection of liver dysfunction, allowing prompt therapeutic interventions such as optimizing fluid balance and initiating supportive therapies to enhance liver recovery.

## 5. Conclusions

This study identified elevated preoperative glucose levels, reduced FLR/SV ratio, and elevated POD 1 TB levels as significant predictors of PHLF. These findings highlight the importance of meticulous preoperative glucose control, volumetric assessment, and early postoperative monitoring to reduce the risk of liver failure. Future studies should focus on validating these predictors in larger populations and exploring targeted interventions to improve patient outcomes after major hepatic resections.

## Figures and Tables

**Table 1:** Preoperative demographics of 34 patients who underwent hepatectomy for Klatskin tumors, comparing those without PHLF or with ISGLS grade A and those with ISGLS grade B or C.

Variable	ISGLS 0/A (*n* = 26)	ISGLS B/C (*n* = 8)	*p*-Value
Age	67.88 (39–81)	73.50 (66–80)	0.263
Sex	18 (69.2%)/8 (30.8%)	5 (62.5%)/3 (37.5%)	1.000
Height (cm)	161.39 (140.6–176.7)	158.70 (145.0–171.0)	0.405
Weight (kg)	58.95 (45.5–76.0)	58.79 (47.0–70.4)	0.968
BMI	22.66 (17.05–29.90)	23.32 (19.56–27.95)	0.676
ASA (1/2/3)	1(3.8%)/20(76.9%)/5(19.2%)	0/3 (37.5%)/5 (62.5%)	0.061
Comorbidity			
HTN	12 (46.2%)	6 (75.0%)	0.306
DM	10 (38.5%)	5 (62.5%)	0.429
HBV	0	0	1.000
HCV	1 (3.7%)	0	1.000
Alcoholism	4 (15.4%)	2 (25.0%)	0.925
Bithmuth type			
II/IIIa/IIIb/IV	1(3.7%)/10(38.5%)/5(19.2%)/10(38.5%)	0/6(75.0%)/1(12.5%)/1(12.5%)	0.488
WBC (µL)	7.01 (2.97–14.3)	8.03 (5.2–12.1)	0.291
Platelet (×10^3^/µL)	26.78 (9.1–39.2)	25.80 (16.6–37.5)	0.477
PT INR	277.81 (140–588)	257.75 (193–308)	0.984
Protein (mg/dL)	0.97 (0.84–1.34)	1.00 (0.91–1.09)	0.246
Albumin (mg/dL)	6.83 (5.3–8.2)	6.72 (6.2–7.1)	0.699
TB (mg/dL)	4.01 (3.0–4.9)	3.86 (3.2–4.6)	0.451
AST (U/L)	0.96 (0.15–3.18)	1.42 (0.57–3.58)	0.138
ALT (U/L)	44.12 (16–228)	33.75 (23–49)	0.871
HS-CRP (mg/dL)	36.31 (6–195)	37.50 (6–70)	0.155
Pre op glucose (mg/dL)	135 (82–296)	178 (135–238)	0.031
APRI	0.46 (0.10–2.30)	0.35 (0.19–0.51)	0.735
ALBI	−2.69 (−3.41–−1.40)	−2.42 (−3.14- −1.70)	0.327
MELD	5.17 (−0.08–11.29)	7.43 (4.28–11.79)	0.064
Rt. HH/Lt. HH	12 (46.2%)/14 (53.8%)	6 (75.0%)/2 (25.0%)	0.306
FLR	696 (136–1407)	544 (129–977)	0.205
spleen	148 (48–350)	199 (115–355)	0.101
FLR/SV	5.23 (1.26–10.76)	2.87 (1.12–6.01)	0.012

PHLF, post-hepatectomy liver failure; ISGLS, The International Study Group for Liver Surgery; BMI, body mass index; ASA, American Society of Anesthesiologists classification; HTN, hypertension; DM, diabetes mellitus; HBV, hepatitis B virus; HCV, hepatitis C virus; WBC, white blood cell; PT, prothrombin time; TB, total bilirubin; AST, aspartate transaminase; ALT, alanine transaminase; HS-CRP, high-sensitivity C-reactive protein; Pre op, preoperative; APRI, AST to Platelet Ratio Index; ALBI, albumin-bilirubin score; MELD, Model for End-Stage Liver Disease score; Rt. HH, right hemi-hepatectomy; Lt. HH, left hemi-hepatectomy; FLR, future liver remnant; FLR/SV, future liver remnant to spleen ratio.

**Table 2:** Multivariable analysis of risk factors for PHLF with preoperative demographics of patients who received hepatectomy d/t Klatskin tumor.

Variable	Odds Ratio	95% CI (Lower–Upper)	*p*-Value
Pre op glucose (mg/dL)	1.03	1–1.06	0.047
FLR/SV	0.382	0.154–0.949	0.038

PHLF, post-hepatectomy liver failure; Pre op, preoperative; FLR/SV, future liver remnant to spleen ratio.

**Table 3:** Immediate postoperative data of 34 patients who underwent hepatectomy for Klatskin tumors, comparing those without PHLF or with ISGLS grade A and those with ISGLS grade B or C.

Variable	ISGLS 0/A (*n* = 26)	ISGLS B/C (*n* = 8)	*p*-Value
OP time (min)	453 (185–655)	539 (405–705)	0.043
EBL (cc)	492 (100–3150)	497 (180–900)	0.296
POD 1 WBC (×10^3^/µL)	14.72 (5.30–24.98)	10.12 (4.95–13.71)	0.011
POD 1 Platelet (×10^3^/µL)	204 (67–439)	206 (109–307)	0.887
POD 1 PT INR	1.20 (1.00–1.50)	1.35 (1.02–2.03)	0.330
POD 1 Protein (mg/dL)	5.51 (3.40–6.30)	5.23 (4.80–6.20)	0.316
POD 1 Albumin (mg/dL)	3.40 (2.40–4.20)	3.34 (2.90–4.10)	0.611
POD 1 TB (mg/dL)	1.49 (0.48–2.83)	3.04 (0.94–5.30)	0.006
POD 1 AST (U/L)	346 (91–2000)	216 (77–340)	0.951
POD 1 ALT (U/L)	245 (38–1112)	180 (18–436)	0.641
POD 1 HS-CRP (mg/dL)	4.54 (0.93–12.54)	7.06 (2.51–14.04)	0.413

PHLF, post-hepatectomy liver failure; Op time, operative time; POD, postoperative; WBC, white blood cell; PT, prothrombin time; TB, total bilirubin; AST, aspartate transaminase; ALT, alanine transaminase; HS-CRP, high-sensitivity C-reactive protein.

**Table 4:** Multivariable analysis of risk factors for PHLF with immediate postoperative data of patients who received hepatectomy d/t Klatskin tumor.

Variable	Odds Ratio	95% CI (Lower–Upper)	*p*-Value
Op time (min)	1.005	0.985–1.024	0.649
POD 1 WBC (×10^3^/µL)	0.609	0.362–1.025	0.062
POD 1 TB (mg/dL)	5.821	1.136–29.818	0.035

PHLF, post-hepatectomy liver failure; Op time, operative time; POD, postoperative; WBC, white blood cell; TB, total bilirubin.

## Data Availability

The data presented in this study are available on request from the corresponding author.

## References

[1] Masunari H., Shimada H., Endo I., Fujii Y., Tanaka K., Sekido H., Togo S. (2008). Surgical anatomy of hepatic hilum with special reference of the plate system and extrahepatic duct. J. Gastrointest. Surg..

[2] Olthof P.B., Wiggers J.K., Groot Koerkamp B., Coelen R.J., Allen P.J., Besselink M.G., Busch O.R., D'Angelica M.I., DeMatteo R.P., Kingham T.P. (2017). Postoperative Liver Failure Risk Score: Identifying Patients with Resectable Perihilar Cholangiocarcinoma Who Can Benefit from Portal Vein Embolization. J. Am. Coll. Surg..

[3] Mullen J.T., Ribero D., Reddy S.K., Donadon M., Zorzi D., Gautam S., Abdalla E.K., Curley S.A., Capussotti L., Clary B.M. (2007). Hepatic insufficiency and mortality in 1059 noncirrhotic patients undergoing major hepatectomy. J. Am. Coll. Surg..

[4] Balzan S., Belghiti J., Farges O., Ogata S., Sauvanet A., Delefosse D., Durand F. (2005). The “50–50 criteria” on postoperative day 5: An accurate predictor of liver failure and death after hepatectomy. Ann. Surg..

[5] Reese T., Gilg S., Böcker J., Wagner K.C., Vali M., Engstrand J., Kern A., Sturesson C., Oldhafer K.J., Sparrelid E. (2024). Impact of the future liver remnant volume before major hepatectomy. Eur. J. Surg. Oncol..

[6] Van den Broek M.A.J., Olde Damink S.W.M., Dejong C.H.C., Lang H., Malagò M., Jalan R., Saner F.H. (2008). Liver failure after partial hepatic resection: Definition, pathophysiology, risk factors and treatment. Liver Int..

[7] Clavien P.A., Petrowsky H., DeOliveira M.L., Graf R. (2007). Strategies for safer liver surgery and partial liver transplantation. N. Engl. J. Med..

[8] Derpapas M.K., Contis J., Fragulidis G.P., Lykoudis P.M., Polymeneas G., Ntourakis S., Voros D. (2013). Correlation of the ICG test with risk factors and postoperative outcomes following hepatic resection. J. BUON.

[9] Park H.J., Seo K.I., Kim S.J., Lee S.U., Yun B.C., Han B.H., Shin D.H., Choi Y.I., Moon H.H. (2021). Effectiveness of Albumin-bilirubin Score as a Predictor of Post-hepatectomy Liver Failure in Patients with Hepatocellular Carcinoma. Korean J. Gastroenterol..

[10] Morandi A., Risaliti M., Montori M., Buccianti S., Bartolini I., Moraldi L. (2023). Predicting Post-Hepatectomy Liver Failure in HCC Patients: A Review of Liver Function Assessment Based on Laboratory Tests Scores. Medicina.

[11] Milana F., Famularo S., Diana M., Mishima K., Reitano E., Cho H.-D., Kim K.-H., Marescaux J., Donadon M., Torzilli G. (2023). How Much Is Enough? A Surgical Perspective on Imaging Modalities to Estimate Function and Volume of the Future Liver Remnant before Hepatic Resection. Diagnostics.

[12] Kim M., Suh S.-W., Lee E.S., Suh S., Lee S.E., Choi Y.S. (2024). Clinical Factors Affecting the Rate of Liver Regeneration in Living Donors after Right Hepatectomy. J. Pers. Med..

[13] Koch D.T., Horné F., Fabritius M.P., Werner J., Ilmer M. (2024). Hepatocellular Carcinoma: The Role of Surgery in Liver Cirrhosis. Visc. Med..

[14] Bucur P.O., Bekheit M., Audebert C., Othman A., Hammad S., Sebagh M., Allard M.A., Decante B., Friebel A., Miquelestorena-Standley E. (2018). Modulating Portal Hemodynamics With Vascular Ring Allows Efficient Regeneration After Partial Hepatectomy in a Porcine Model. Ann. Surg..

[15] Regimbeau J.M., Kianmanesh R., Farges O., Dondero F., Sauvanet A., Belghiti J. (2002). Extent of liver resection influences the utcome in patients with cirrhosis and small hepatocellular carcinoma. Surgery.

[16] Bae J.S., Lee D.H., Yoo J., Yi N.J., Lee K.W., Suh K.S., Kim H., Lee K.B. (2021). Association between spleen volume and the post-hepatectomy liver failure and overall survival of patients with hepatocellular carcinoma after resection. Eur. Radiol..

[17] Oh N., Kim J.H., Rhu J., Jeong W.K., Choi G.S., Kim J.M., Joh J.W. (2023). Automated 3D liver segmentation from hepatobiliary phase MRI for enhanced preoperative planning. Sci. Rep..

[18] Oh N., Kim J.H., Rhu J., Jeong W.K., Choi G.S., Kim J.M., Joh J.W. (2024). 3D auto-segmentation of biliary structure of living liver donors using magnetic resonance cholangiopancreatography for enhanced preoperative planning. Int. J. Surg..

[19] Oh N., Kim J.H., Rhu J., Jeong W.K., Choi G.S., Kim J.M., Joh J.W. (2024). Comprehensive deep learning-based assessment of living liver donor CT angiography: From vascular segmentation to volumetric analysis. Int. J. Surg..

[20] Rahbari N.N., Garden O.J., Padbury R., Brooke-Smith M., Crawford M., Adam R., Koch M., Makuuchi M., Dematteo R.P., Christophi C. (2011). Posthepatectomy liver failure: A definition and grading by the International Study Group of Liver Surgery (ISGLS). Surgery.

[21] Wang Q., Wei S., Zhou H., Shen G., Gan X., Zhou S., Qiu J., Shi C., Lu L. (2019). Hyperglycemia exacerbates acetaminophen-induced acute liver injury by promoting liver-resident macrophage proinflammatory response via AMPK/PI3K/AKT-mediated oxidative stress. Cell Death Discov..

[22] Mendes-Braz M., Martins J.O. (2018). Diabetes Mellitus and Liver Surgery: The Effect of Diabetes on Oxidative Stress and Inflammation. Mediat. Inflamm..

[23] Søreide J.A., Deshpande R. (2021). Post hepatectomy liver failure (PHLF)—Recent advances in prevention and clinical management. Eur. J. Surg. Oncol..

[24] Piccus R., Joshi K., Hodson J., Bartlett D., Chatzizacharias N., Dasari B., Isaac J., Marudanayagam R., Mirza D.F., Roberts J.K. (2023). Significance of predicted future liver remnant volume on liver failure risk after major hepatectomy: A case matched comparative study. Front. Surg..

[25] Yu Q., Xu C., Li Q., Ding Z., Lv Y., Liu C., Huang Y., Zhou J., Huang S., Xia C. (2022). Spleen volume-based non-invasive tool for predicting hepatic decompensation in people with compensated cirrhosis (CHESS1701). JHEP Rep..

[26] Romero-Cristóbal M., Clemente-Sánchez A., Ramón E., Téllez L., Canales E., Ortega-Lobete O., Velilla-Aparicio E., Catalina M.V., Ibáñez-Samaniego L., Alonso S. (2022). CT-derived liver and spleen volume accurately diagnose clinically significant portal hypertension in patients with hepatocellular carcinoma. JHEP Rep..

[27] Ito T., Tanemura A., Kuramitsu T., Murase T., Kaluba B., Noguchi D., Fujii T., Yuge T., Maeda K., Hayasaki A. (2023). Spleen volume is a predictor of posthepatectomy liver failure and short-term mortality for hepatocellular carcinoma. Langenbeck’s Arch. Surg..

[28] May B.J., Madoff D.C. (2012). Portal vein embolization: Rationale, technique, and current application. Semin. Interv. Radiol..

[29] Chan A., Zhang W.Y., Chok K., Dai J., Ji R., Kwan C., Man N., Poon R., Lo C.M. (2021). ALPPS Versus Portal Vein Embolization for Hepatitis-related Hepatocellular Carcinoma: A Changing Paradigm in Modulation of Future Liver Remnant Before Major Hepatectomy. Ann. Surg..

[30] Kudo M., Gotohda N., Sugimoto M., Konishi M., Takahashi S., Kobayashi S., Kobayashi T. (2022). The Assessment of Regional Liver Function Before Major Hepatectomy Using Magnetic Resonance Imaging. Am. Surg..

[31] Etra J.W., Squires M.H., Fisher S.B., Rutz D.R., Martin B.M., Kooby D.A., Cardona K., Sarmiento J.M., Staley C.A., Maithel S.K. (2014). Early identification of patients at increased risk for hepatic insufficiency, complications and mortality after major hepatectomy. HPB.

[32] Sawangkajohn W., Luvria V., Leeratanakachorn N., Tipwaratorn T., Theerakul S., Jarearnrat A., Titapun A., Srisuk T., Pugkhem A., Khuntikeo N. (2020). Re-Rising of Total Bilirubin Level after Postoperative Day 3 (The V Pattern) Predicting Liver Failure and Survival of Patients who Underwent Hepatectomy for Cholangiocarcinoma. Asian Pac. J. Cancer Prev..

[33] Jones R.P., Vauthey J.N., Adam R., Rees M., Berry D., Jackson R., Grimes N., Fenwick S.W., Poston G.J., Malik H.Z. (2012). Effect of specialist decision-making on treatment strategies for colorectal liver metastases. Br. J. Surg..

[34] Kang C.M., Ku H.J., Moon H.H., Kim S.-E., Jo J.H., Choi Y.I., Shin D.H. (2024). Predicting Safe Liver Resection Volume for Major Hepatectomy Using Artificial Intelligence. J. Clin. Med..

